# Curcumin-Loaded Pickering Emulsion Formed by Ultrasound and Stabilized by Metal Organic Framework Optimization

**DOI:** 10.3390/foods10030523

**Published:** 2021-03-03

**Authors:** Peihua Ma, Zhi Zhang, Shawn Tsai, Hongchao Zhang, Yuan Li, Fang Yuan, Qin Wang

**Affiliations:** 1 Department of Nutrition and Food Science, College of Agriculture and Natural Resources, University of Maryland, College Park, MD 20740, USA; peihua@umd.edu (P.M.); zzhangkk@terpmail.umd.edu (Z.Z.); stsai12@umd.edu (S.T.); hzhang18umd@outlook.com (H.Z.); liyuan19930603@outlook.com (Y.L.); 2 Beijing Advanced Innovation Center for Food Nutrition and Human Health, Beijing Laboratory for Food Quality and Safety, College of Food Science and Nutritional Engineering, China Agricultural University, Beijing 100083, China; yuanfang0220@cau.edu.cn

**Keywords:** metal-organic framework, Pickering emulsion, curcumin, response surface methodology, ultrasonic encapsulation

## Abstract

The ultrasound-assisted preparation of a curcumin-loaded metal organic framework (MOF) UiO-66-NH_2_ stabilized Pickering emulsion system was carried out in this study. A 3-level-4-factor Box–Behnken design (BBD) and response surface methodology (RSM) analysis were employed to systematically evaluate the effect of different experimental parameters (i.e., ultrasonic power, ultrasonic time, oil content, and MOF content) on curcumin loading capacity (LC) and encapsulation efficiency (EE). The results indicated that ultrasonic power and MOF content significantly affected LC and EE, whereas ultrasonic time and oil content had little effect. A mathematical model for optimizing the preparation of emulsion systems was established. Based on the ridge max analysis, an optimal condition for the newly developed curcumin-loaded MOF-Pickering emulsion was identified, i.e., ultrasonic power 150 W, ultrasonic time 11.17 min, oil content 20.0%, and MOF content 1.10%. At this condition, the LC and EE of curcumin obtained from the experiment reached 7.33% ± 0.54% and 56.18% ± 3.03%, respectively, which were within the prediction range of LC (7.35% ± 0.29%) and EE (54.34% ± 2.45%). The emulsion systems created in this study may find new applications for the delivery of bioactive compounds in food and pharmaceutical areas.

## 1. Introduction

Ultrasound is widely applied in cell disruption, chemical syntheses, extraction, and emulsification in the food industry because it can homogenize and generate a high-viscosity system with a uniform droplet size distribution [1]. The versatility of ultrasound refers to its wide effective frequency region that makes it possible to control the cavitation intensity and the number of cavitation events of the system [2]. The mechanism of ultrasound homogenization has been illustrated as follows: high-energy ultrasonic waves generate cavitation in a liquid solution, and subsequent collapses of the cavitation bubbles are facilitated by stirring and thorough mixing of the solution [2]. The ultrasonic method has been proven effective at preparing highly stable emulsions, polymeric particles with environmental sensitivity (i.e., sensitive to light, heat, and pressure), and microspheres for encapsulating bioactive compounds [3].

The ultrasonic-assisted preparation of Pickering emulsion systems has attracted attention recently. Pickering emulsions comprise a special category of emulsions stabilized by solid particles of emulsifiers located at the oil–water interface. They were discovered a century ago, but have only been studied extensively recently [4]. When compared to traditional emulsion systems, Pickering emulsions usually have a better stability since solid particles at the interface prevent the emulsion’s coalescence. Previous studies have used ultrasonic-assisted homogenization to prepare Pickering emulsions from solid particles with various origins, for example, polyaniline/nano-ZnO composites, Fe_3_O_4_ nanoparticles, and cellulose nanocrystals [1,2,5]. 

On the other hand, metal organic frameworks (MOFs), a class of hybrid porous materials with uniform structures, have been studied due to their high porosity and tunable physical and chemical properties [6], and have the potential to be used as stabilizers for Pickering emulsions. When they were first introduced, the application of MOFs was limited to the catalysis, separation, and storage of gas mixtures, whereas their application has been expanded to include hazardous material adsorption, new catalysis, and biomedical applications [7]. To date, MOFs have demonstrated their ability to be specifically tailored toward different properties and have been reported for application in many food sectors, including food packaging, contaminant detection, and antibacterial delivery [8]. Meanwhile, with an in-depth understanding of MOFs as a porous material, low-toxicity MOFs have been designed as drug delivery vehicles and tissue imaging agents [9]. Some MOFs have been found to show few adverse effects in the host during tests, and display a good biocompatibility [10]. Since the first structure was reported in 1976, more than 80,000 MOF structures were added in the Cambridge Crystallographic Data Centre (CCDC), known as the world’s repository of small-molecule crystal structures, and the number of collates increased dramatically in the past decade [11,12]. The simplest MOF structure is in the form of 1,4-benzene dicarboxylic acid (BDC) linkers connected to zinc, copper, or zirconium salts/oxides by a carboxyl group. The well-known examples are IRMOF-1, HKUST-1, and UiO-66 and their derivatives [13,14]. Among them, UiO-66 and its derivatives are usually used as model materials for studying MOF structures because of their facile synthesis, simple structures, and typical physicochemical properties. As model MOFs, the UiO-66 family, has been applied to many aspects of research, including but not limited to the use of synthesis control, group modification, toxicology evaluation, and delivery systems [15]. In recent years, many reports utilized MOFs as delivery systems due to their high porosity. Meanwhile, due to the strong surface charge of MOF particles, they can be utilized to prepare stable emulsions preventing droplet coalescence and aggregation, but the research on applying MOFs as stabilizers for Pickering emulsions is still in an early stage [16]. 

In general, emulsion systems stabilized by solid particles (e.g., SiO_2_ and proteins) instead of molecular surfactants are recognized as “Pickering emulsions” [17]. Compared to conventional emulsion systems, Pickering emulsions have shown desirable characteristics; for example, a lower amount of emulsifier is needed and better stability can be achieved by preventing coalescence from happening, which facilitates their application in the cosmetics, pharmaceutical, and food industries [18]. Moreover, these Pickering emulsion systems could be further developed to delivery systems for encapsulating bioactive components (e.g., curcumin) [19]. Curcumin, a representative phytochemical compound, has been reported for numerous benefits, including cancer prevention, anti-inflammation, and neuroprotection [20]. However, the low water solubility, photosensitivity, and poor oral bioaccessibility of curcumin has seriously limited its applications as an attractive food colorant or a dietary supplement [20,21]. To address this challenge, various delivery systems have been developed and demonstrated to retain its biological functions and expand its applicability [22]. Encapsulating lipophilic curcumin with Pickering-emulsion-based delivery systems has proven to be a useful means to surmount the disadvantages of curcumin [23].

The exploration of novel, stable, and functional Pickering emulsion systems using different stabilizing materials has been a hot topic of research in past decades. In this study, for the first time, we report an ultrasound-assisted MOF-Pickering emulsion system for encapsulating curcumin (a representative bioactive compound for testing Pickering emulsion characteristics). Medium-chain triglycerides (MCTs) were selected as a dispersed phase, UiO-66-NH_2_ was used as the stabilizer, and curcumin was loaded in the MCTs with an ultrasonic solubilization. The effect of experimental conditions (i.e., ultrasonic power, ultrasonic time, oil content, and MOF content) on the responsive results (i.e., loading capacity and encapsulation efficiency) was analyzed using a Box–Behnken design (BBD) and response surface methodology (RSM). Attempts were made to obtain optimal conditions for the curcumin-loaded UiO-66-NH_2_ stabilized Pickering emulsion.

## 2. Materials and Methods

### 2.1. Materials

Zirconium tetrachloride (ZrCl_4_, 99.5%), BDC (98.0%), N,N-dimethylformamide (DMF, 99.0%), ethyl alcohol (99.5%), polyethylene glycol (PEG, m.w. 10,000 Da), curcumin (99.5%), medium-chain triglyceride (MCT), and acetic acid (99.9%) were purchased from Sigma-Aldrich (St. Louis, MO, USA). All of the chemicals were analytical grade.

### 2.2. Experimental Design

A 3-level-4-factor BBD, including 29 experiments, was employed in this study. To avoid bias, 29 runs were performed in a random order. The variables and their levels selected for the preparation of the MOF-stabilized emulsions were: ultrasonic power 50–150 W, ultrasonic time 5–15 min, MCTs content 15–30% *w*/*w*, and UiO-66-NH_2_ content 1–3% *w*/*w*. All experiments were performed in a 30 g system. Table 1 shows the independent factors (X_i_) and their levels.

### 2.3. Synthesis of UiO-66-NH_2_

The UiO-66-NH_2_ was synthesized according to a previous report with a minor modification [24]. First, ZrCl_4_ (1.17 g, 5 mmol), BDC (0.90 g, 5 mmol), and acetic acid (1.0 mL) were dissolved in DMF (30 mL) at room temperature. Then, the mixture was placed in a Teflon-lined stainless-steel autoclave after adding 2 mL of de-ionized (DI) water and mixed completely. The autoclave was heated in an oil bath at 120 °C for 24 h. Afterward, the solution was cooled down at room temperature for 30 min, and the resulting UiO-66-NH_2_ particles were separated via centrifugation (12,096× *g*, 10 min) at room temperature and washed three times with ethanol. The resulting white powder was obtained by drying the particles in an oven for 24 h [24]. The particle size of synthesized UiO-66-NH_2_ was 161.36 nm, measured by dynamic light scattering (BI-200SM, Brookhaven Instruments Corp., Holtsville, NY, USA) with crystal structure verified by an X-ray diffraction (XRD) diffractometer (C2 Discover Bruker Diffractometer, Madison, WI, USA) as shown in Figure S1.

### 2.4. Emulsion Preparation

Ultrasound can be categorized into three different regions along the frequency spectrum: the power ultrasound region (16–100 kHz), the intermediate ultrasound region (100–1000 kHz), and the ultra-high-frequency ultrasound region (above 1000 kHz) [3]. In the power ultrasound region used in this study, the radius of cavitation bubbles during homogenization can be calculated using Equation (1):

(1) $$R_{r}=\sqrt{\frac{3ϒp_{\infty }}{\rho_{L}\omega^{2}}}$$

where ϒ is the specific heat ratio of the gas [15] inside the bubble, *p*_∞_ is the ambient liquid pressure, *ρ*_L_ is the liquid density, and ω is the angular frequency of ultrasound. In practice, the size of an active bubble is expressed as *R_r_* ≈ 3/*F*, where *F* is the frequency of the ultrasound. In the power ultrasound region, ultrasound delivers high energy into the solution, converting it to localized shear force and increased temperature. The energy density of the solution can be as high as 1000 W/cm^2^, and is directly influenced by the input power [25].

Briefly, 1.5 g of curcumin was dissolved in 200 mL MCTs as a stock solution, and it was treated with ultrasound (Model 505, Fisherbrand, PA, USA) in the conditions of a 1 s interval and under 20 kHz, 390 W for 30 min. After centrifugation, the supernatant was collected as the oil phase (dispersed phase). The parameters for the preparation of the curcumin-loaded emulsion were optimized in our previous report [26]. The mixtures with different concentrations of UiO-66-NH_2_ with 10% *w*/*w* PEG (as a depleter) in water were vortexed and then treated with ultrasound in the conditions of a 1 s interval and 390 W for 15 min. The solution was kept at room temperature for 24 h as the continuous phase. Oil-in-water (O/W) Pickering emulsions were prepared as follows. Different amounts of dispersed phase were added into the continuous phase. After that, mixtures were sheared to form coarse emulsions using a high-speed homogenizer (Ultra-Turrax T25, IKA, Staufen, Germany) at 24,000 rpm for 5 min. The obtained coarse emulsions were treated with high-energy ultrasonic waves at different powers (50–150 W) for different periods of time (5–15 min) using a sonic dismembrator (Model 505, Fisherbrand, PA, USA) in an ice bath. Each system had a total mass of 30 g [27].

### 2.5. Encapsulation Properties

The content of curcumin within the Pickering emulsions was analyzed using the method reported in [28]. Briefly, curcumin was extracted by MCTs with the ultrasonic-assisted method, then its concentration was obtained from a standard curve using a UV−visible spectrophotometer (Beckman Coulter, Brea, CA, USA) at 426 nm (curcumin content in MCT stock was 10.07 mg/L). The loading capacity (LC) and encapsulation efficiency (EE) of curcumin were calculated by Equations (2) and (3), respectively:

(2) $$LC=\frac{Encapsulated\;curcumin}{Total\;mass\;of\;Pickering\;emulsion}\;\times 100\%,$$

(3) $$EE=\frac{Encapsulated\;curcumin}{Total\;curcumin}\;\times 100\%.$$

### 2.6. Statistical Analysis

All data were obtained in triplicate and values were expressed as the mean ± standard deviation. The experimental data (Table 1) were analyzed by response surface regression (RSREG) using the SAS v9.3 software to fit the following second-order polynomial Equation (4):

(4) $$Y=b_{0}+\sum_{i=1}^{4}b_{i}X_{i}+\sum_{i=1}^{4}b_{ii}X_{i}^{2}+\sum_{i=1}^{3}\sum_{j=i+1}^{4}b_{ij}X_{i}X_{j},$$

where *Y* is the response (percent of molar conversion); *b*_0_ is a constant, *b_i_*, *b_ii_*, and *b_ij_* are coefficients; *X_i_* and *X_j_* are the uncoded independent variables. The options of RSREG SAS and RIDGE MAX were employed to compute the estimated ridge of maximum response for increasing radii from the center of the original design [29]. The significant level (***p***) was set at 0.05.

## 3. Results and Discussion

In order to systemically investigate and optimize the influence of ultrasonic power, ultrasonic time, oil content, and UiO-66-NH_2_ content on LC and EE of the Pickering emulsions, a 3-level-4-factor BBD was applied, and a total of 29 treatments were carried out. RSM was then applied to analyze the experimental data. The results of the curcumin LC and EE are shown in Table 1. Of the total 29 treatments, treatment #4 (ultrasonic power 100 W, ultrasonic time 15 min, oil content 15.0%, and MOF content 2%) resulted in the highest LC (7.01% ± 0.41%) and treatment #21 (150 W ultrasonic power, 10 min ultrasonic time, 22.5% oil content, and 1% MOF content) resulted in the highest EE (58.41% ± 0.95%), whereas treatment #6 (ultrasonic power 100 W, ultrasonic time 10 min, oil content 30.0%, and MOF content 1%) resulted in the lowest LC (1.61% ± 0.11%) and treatment #11 (ultrasonic power 100 W, ultrasonic time 10 min, oil content 15.0%, and MOF content 3%) resulted in the lowest EE (17.86% ± 3.17%).

In addition, the RSREG procedure, which calculates the least squares to fit quadratic response surface regression models from SAS, was employed to fit the second-order polynomial. Equations (5) and (6) were thus generated and are given as below:

(5) $$Y_{LC}\left(\%\right)=\left(0.0440+0.0172X_{1}+0.2419X_{2}+0.2901X_{3}+1.2325X_{4}-0.0051X_{2}X_{3}\;+0.0174X_{2}X_{4}-0.0062X_{2}^{2}-0.0062X_{3}^{2}-0.3910X_{4}^{2}\right)\times 100\%$$

(6) $$Y_{EE}\left(\%\right)=\left(0.6352+0.1263X_{1}+1.5573X_{2}+1.5314X_{3}-47.1652X_{4}-0.0168X_{1}X_{4}\;-0.0305X_{2}X_{3}+0.2027X_{3}X_{4}-0.0406X_{2}^{2}+6.6207X_{4}^{2}\right)\times 100\%$$

Analysis of variance (Table 2) indicated that this quadratic polynomial model was highly significant and adequate to represent the actual relationship between variables and response factors. For LC, the *p*-value was <0.0001, and R^2^ was 0.9017. For EE, the *p*-value was <0.0001, and R^2^ was 0.9965.

A joint test was further used to analyze the overall effect of four synthesis variables on LC and EE. As shown in Table 3, ultrasonic power (X_1_) affected LC significantly. Meanwhile, ultrasonic power (X_1_) and MOF content (X_4_) in the range of 50–150 W and 1–3% *w*/*w*, respectively, affected the EE significantly (*p* < 0.1). Moreover, the *p*-value of MOF content (X_4_) for LC and oil content (X_3_) for EE was only slightly higher than 0.1, with a non-negligible improvement of the estimated results. Ultrasonic time (X_2_) did not show a significant effect on the LC and EE.

### 3.1. Mutual Effect of Parameters

**Figure 1:**
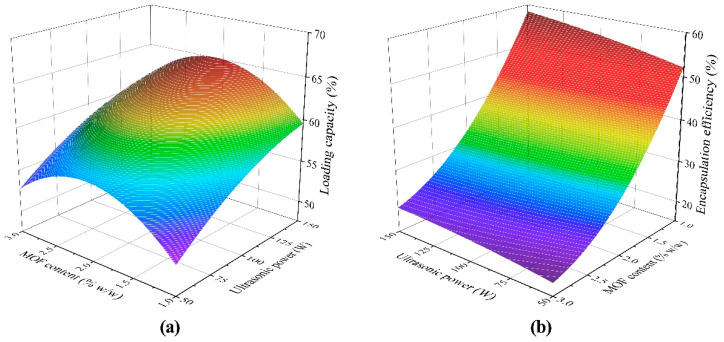
Response surface plot showing the effect of ultrasonic power and MOF content on (**a**) loading capacity (LC); (**b**) encapsulation efficiency (EE).

The response surface plots were used to investigate the mutual effects of ultrasonic power and MOF content on the LC and EE. The results from the BBD experiment were plotted with the ultrasonic time and oil content set at 10 min and 22.5% *w*/*w*, respectively. Figure 1a,b shows the effect of the ultrasonic power ranging from 50 to 150 W, and MOF content from 1.0% to 3.0% *w*/*w* on LC and EE, respectively. LC increased continuously with increasing ultrasonic power. However, with increasing MOF content from 1.0% to 3.0% *w*/*w*, LC reached a maximum value at 2.0% *w*/*w*. On the other hand, the trends of MOF content and ultrasonic power to EE were both monotonic. To sum up, it appears that increasing ultrasonic power led to higher LC and EE, but increasing MOF content caused the LC to have a peak value, and EE decreased continuously as MOF content increased.

In addition to the qualitative prediction for the four factors, the statistical analysis results can be seen in Table 4. For both LC and EE, the *p*-values for ultrasonic power are less than 0.0001. MOF content comes in the second position, while ultrasonic time and oil content do not appear as significant. The estimated independent weight of both LC and EE models share a similar trend, except for a few minor interaction differences.

### 3.2. Optimization of Synthesis Conditions

For the ultrasonic power factor, upsurging ultrasonic power led to a monotonous increase for both LC and EE. This result indicates that for the preparation of a Pickering emulsion by ultrasound waves, within a relatively wide range, increasing the ultrasonic power can significantly improve the LC and EE of the delivery system. This can be explained by the formation of cavitation bubbles during ultrasonic homogenization. This phenomenon led to the strong correlation between droplet particle size and ultrasonic power, and this result is consistent with previous reports [15,30].

However, it should be noted that increasing the ultrasound power to a threshold value will cause the local temperature of the emulsion system to increase quickly during the ultrasound process, which may lead to the oxidation of natural products. In a preliminary test we observed that when the ultrasonic power level was >150 W, the system temperature was not easily maintained during the emulsion preparation, even with an ice-water bath. Therefore, we selected the 150 W power level as the upper limit for the BBD modeling.

Compared to the ultrasonic power, extended ultrasonic time did not have a significant impact on LC and EE. This indicates that the Pickering emulsion had already been formed or entered a homogeneous steady state after 10 min of ultrasonic treatment. According to our previous study, the droplet size of MOF-stabilized Pickering systems was attributed to competition for MOF particles between the continuous phase and droplet interface. This competition resulted in an equilibrium of MOF concentration in the continuous phase and at the interface of droplets. The LC and EE are related to the droplet size. After the homogenization exceeded 10 min, the LC and EE of the emulsion were no longer related to the ultrasonic treatment time because both the amount of MOF at the interface and droplet size reached a dynamic equilibrium.

**Figure 2:**
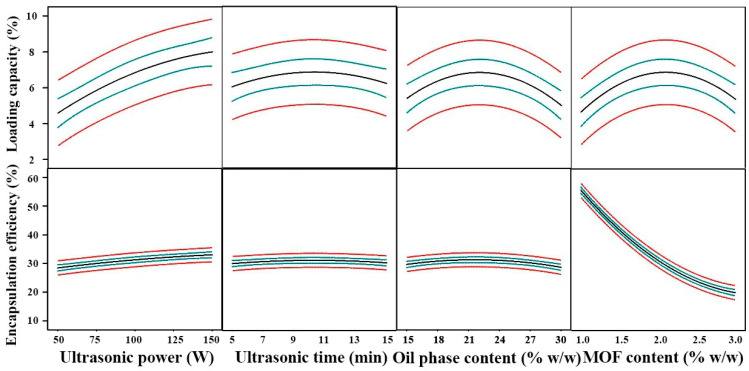
Prevision profiler’s outputs after maximization of the desirability function for LC and EE: Optimum emulsion preparation conditions are given, while curves show how changes of factors’ levels affect the response. Green lines indicate 95% confidence intervals and red lines indicate model predictions.

Similar to the ultrasonic time, LC and EE had peak values when the oil content reached around 22.50% *w*/*w* (Figure 2). Unlike the previous three independent variables, the investigation of optimal conditions for MOF content was the most complicated. For LC, the optimal value of MOF content appeared around 2.0% *w*/*w*. Nevertheless, for EE, as the MOF content increased within the tested range, EE decreased monotonically. It is easy to understand that, by definition, LC has no direct relationship with MOF content as shown in Equation (2), but for the EE, MOF content itself is the denominator in Equation (2). Therefore, in order to eliminate interference, we used LC as the response for MOF content optimization.

The optimum conditions for preparing MOF-Pickering emulsion systems was determined by ridge max analysis, which computes the estimated ridge of maximum response for increasing radii from the center of the original design. Figure 2 shows the determination. The highest values of LC (7.35% ± 0.29%) and EE (54.34% ± 2.45%) were predicted at the following conditions: 150 W ultrasonic power, 11.17 min ultrasonic time, 20.0% *w*/*w* oil content, and 1.10% *w*/*w* MOF content.

### 3.3. Model Validation

**Figure 3:**
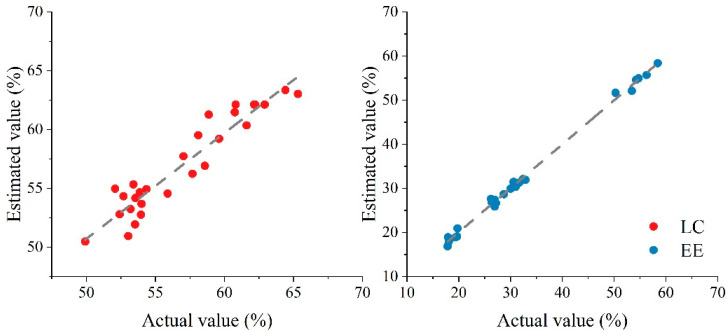
Validation of the model with experimental data.

We used the slope of a regression line to evaluate the similarity between experimental observation and model prediction. The scatter plots of validation data are shown in Figure 3, where the *x*-axis indicates the values from experiments while the *y*-axis indicates the values from model simulation, and a slope value closer to 1 indicates a better regression. In addition, a narrower distribution of data points also means that the model is more effective. In Figure 3, the data points between the actual and estimated values of LC and EE show significant linear relationships without obvious outliers. Meanwhile, compared with LC, the data point distribution for EE is narrower, which means the model is more effective. This result was further confirmed with the R^2^ value in Table 2.

Optimum conditions are usually used to validate the model equation for prediction of optimal responses. The validation adequacy of the predicted model (Equations (5) and (6)) was examined by a delivery system that was prepared under the aforementioned optimal conditions. The results showed that LC and EE were respectively 7.33% ± 0.54% and 56.18% ± 3.03% under the optimal conditions. This indicates that LC and EE did not significantly differ from the predicted LC (7.35% ± 0.29%) and EE (54.34% ± 2.45%). Thus, the developed model, as shown in Equations (5) and (6), adequately predicted the LC and EE for preparing the curcumin-loaded MOF-Pickering emulsion delivery systems.

## 4. Conclusions

The optimization of ultrasound in curcumin-loaded Pickering emulsions with UiO-66-NH_2_ as a stabilizer was investigated. A 3-level-4-factor BBD experiment and the RSREG procedure from SAS was employed for the experimental design and data analysis, respectively. Four parameters (i.e., ultrasonic power, ultrasonic time, oil content, and MOF content) were evaluated. According to the response surface analysis, ultrasonic power and MOF content significantly affected the LC and EE (*p* < 0.0001), whereas ultrasonic time and oil content did not. The experimental data were used to establish a model for the preparation of the MOF-Pickering emulsion systems, and optimal preparation parameters were deduced from the model. The optimized conditions were found at an ultrasonic power of 150 W, ultrasonic time of 11.17 min, oil content of 20.0% *w*/*w*, and MOF content of 1.10% *w*/*w*. Furthermore, a Pickering emulsion delivery system was prepared under these optimal conditions, and LC and EE of 7.33% ± 0.54% and 56.18% ± 3.03%, respectively, were obtained for curcumin. Validation tests proved the optimization parameters of this ultrasound-assisted preparation of the curcumin-loaded Pickering emulsion delivery system; LC and EE matched well with the RSM prediction. Therefore, the optimization procedure was considered successful. More research needs to be carried out for evaluating the toxicity of MOFs to better use them in food systems. Future study will focus on the storage stability of MOF delivery systems, and will investigate the interfacial stabilization mechanism using porous materials as stabilizers. The newly developed delivery system in this study may find applications for the delivery of bioactive agents in functional foods and food safety.

## Figures and Tables

**Table 1:** Box–Behnken design (3-level-4-factor) for curcumin-loaded Pickering emulsion delivery system and response surface analysis of experimental data.

No.	Factors				Responses			
	Ultrasonic Power (W)	Ultrasonic Power (min)	Oil Content (% *w*/*w*)	MOF * Content (% *w*/*w*)	Loading Capacity (%)	S.D. *	Encapsulation Efficiency (%)	S.D. *
1	100	10	30	3	4.86	0.38	17.93	0.10
2	100	5	22.5	3	6.79	0.23	19.36	4.78
3	150	5	22.5	2	5.33	0.30	32.89	1.48
4	100	15	15	2	7.01	0.41	30.01	0.30
5	100	15	22.5	1	2.31	0.17	54.73	1.45
6	100	10	30	1	1.61	0.11	50.27	4.28
7	100	5	30	2	3.56	0.23	28.71	3.19
8	50	5	22.5	2	4.30	0.18	26.53	3.79
9	150	10	22.5	3	6.93	0.41	19.76	3.84
10	100	10	22.5	2	5.05	0.34	31.17	3.21
11	100	10	15	3	8.87	0.57	17.86	4.39
12	150	10	30	2	3.85	0.05	31.02	4.86
13	100	10	22.5	2	4.96	0.03	30.62	4.11
14	100	10	22.5	2	5.10	0.16	31.46	0.73
15	150	10	15	2	7.15	0.57	30.59	0.67
16	100	15	22.5	3	6.89	0.02	19.67	3.06
17	50	10	22.5	1	2.26	0.14	53.41	3.17
18	100	10	22.5	2	5.14	0.16	31.68	4.91
19	100	5	15	2	6.30	0.41	26.96	2.80
20	100	15	30	2	3.38	0.17	27.19	3.50
21	150	10	22.5	1	2.47	0.04	58.41	0.59
22	50	10	30	2	3.35	0.25	26.95	3.56
23	50	15	22.5	2	4.25	0.35	26.22	3.82
24	50	10	15	2	6.17	0.13	26.39	0.25
25	150	15	22.5	2	5.26	0.40	32.43	4.40
26	50	10	22.5	3	6.35	0.40	18.11	2.46
27	100	5	22.5	1	2.29	0.17	54.24	0.92
28	100	10	15	1	3.49	0.26	56.28	3.08
29	100	10	22.5	2	5.08	0.13	31.33	3.65

* MOF stands for metal organic framework. S.D. stands for standard deviation.

**Table 2:** Analysis of variances for the variables in preparation of the Pickering emulsion pertaining to response loading capacity (LC) and encapsulation efficiency (EE).

Response Factor	Model	Degree of Freedom	*p*-Value	Std. Deviation	R^2^
LC	Linear	20	0.0056	3.4853	0.4432
	2FI	14	0.9022	3.8099	0.5009
	Quadratic	10	<0.0001	1.9172	0.9017
	Cubic	2	0.0138	0.9031	0.9906
EE	Linear	20	<0.0001	4.1877	0.9027
	2FI	14	0.9911	4.7351	0.9067
	Quadratic	10	<0.0001	1.0366	0.9965
	Cubic	2	0.0066	0.4280	0.9997

**Table 3:** Analysis of variances for preparation of the delivery system by a joint test.

Response Factor	Factor	Sum of Squares	*p*-Value
LC	Ultrasonic Power (X_1_)	18.5340	5.0422
	Ultrasonic Time (X_2_)	0.6960	0.19
	Oil Phase Concentration (X_3_)	2.9304	0.8
	MOF Concentration (X_4_)	9.4696	2.58
EE	Ultrasonic Power (X_1_)	62.98	<0.0001
	Ultrasonic Time (X_2_)	0.2	0.6726
	Oil Phase Concentration (X_3_)	3.01	0.1164
	MOF Concentration (X_4_)	3839.55	<0.0001

**Table 4:** Model parameter coefficients (and their associated *p*-value between brackets) for the prediction of the LC and EE of curcumin. Coefficients have to be used with coded levels for each factor.

	LC		EE	
Model Parameters	Coefficient Estimate	*p*-Value	Coefficient Estimate	*p*-Value
X_1_	0.0172	<0.0001	0.1263	<0.0001
X_2_	0.2419	NS	1.5573	NS
X_3_	0.2901	NS	1.5314	0.1164
X_4_	1.2325	0.1308 ^	−47.1652	<0.0001
X_1_*X_2_	−0.0003	NS	−0.0002	NS
X_1_*X_3_	−0.0002	NS	−0.0001	NS
X_1_*X_4_	−0.0003	NS	−0.0168	0.1285 ^
X_2_*X_3_	−0.0051	0.0327	−0.0305	0.0443
X_2_*X_4_	0.0174	NS	−0.0090	NS
X_3_*X_4_	0.2057	0.1299 ^	0.2027	0.0109
X_1_*X_1_	−0.0006	0.0879	−0.0002	NS
X_2_*X_2_	−0.0062	0.0298	−0.0406	NS
X_3_*X_3_	−0.0062	<0.0001	−0.0375	0.0001
X_4_*X_4_	−0.3910	<0.0001	6.6207	<0.0001

^ These parameters were kept because their coefficient *p*-value was only slightly higher than 0.1, with non-negligible improvement of the estimated result. NS: Non-significant parameters (*p* < 0.1).

## Data Availability

Data is contained within the article or supplementary material.

## Supplementary Materials

The following are available online at https://www.mdpi.com/2304-8158/10/3/523/s1, Figure S1: (**a**) Scanning electron microscope (SEM) images of UiO-66-NH_2_; (**b**) Particle X-ray diffraction (PXRD) patterns of UiO-66-NH_2_.
