# Supplementary material for: Curcumin-Loaded Pickering Emulsion Formed by Ultrasound and Stabilized by Metal Organic Framework Optimization
Source: Foods. 2021 Mar 3;10(3):523. doi: 10.3390/foods10030523 (PMC7998958; doi:10.3390/foods10030523)
Supplement: Supplementary file 1 [file foods-10-00523-s001.pdf]

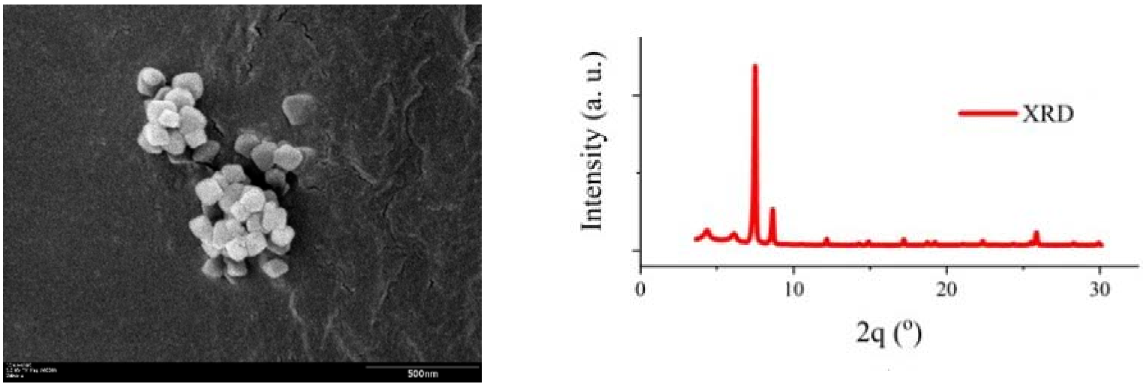

(**a**) (**b**)

Figure S1. (**a**) Scanning electron microscope (SEM) images of UiO-66-NH_2; (**b**) Particle X-ray diffraction (PXRD) patterns of UiO-66-NH_2.
